# Leukocyte Telomere Length Predicts Progression From Paroxysmal to Persistent Atrial Fibrillation in the Long Term After Catheter Ablation

**DOI:** 10.3389/fcvm.2021.813390

**Published:** 2022-01-24

**Authors:** Qianhui Wang, Zheng Liu, Ying Dong, Xinchun Yang, Mulei Chen, Yuanfeng Gao

**Affiliations:** Heart Center and Beijing Key Laboratory of Hypertension, Beijing Chaoyang Hospital, Capital Medical University, Beijing, China

**Keywords:** telomere length, atrial fibrillation, progression, biomarker, catheter ablation

## Abstract

**Background:**

Aging is significantly associated with the incidence and progression of atrial fibrillation (AF) incidence. This study aimed to evaluate the potential predictive value of leukocyte telomere length (LTL) for progression from paroxysmal AF (PAF) to persistent AF (PsAF) after catheter ablation.

**Methods and Results:**

A total of 269 patients with AF (154 patients with PAF and 115 patients with PsAF, respectively) were prospectively enrolled, and all patients with PAF at baseline were regularly followed up to determine whether and when they should progress to PsAF after catheter ablation therapy. Baseline relative LTL was measured by quantitative real-time PCR (rt-PCT). There was a significant negative association between LTL and age (*r* = −0.23, *p* < 0.001). Patients with PsAF had significantly shorter LTL than those with PAF. After a mean follow-up of 854.9 ± 18.7 d, progression events occurred in 35 out of the 154 patients with PAF. Those progressed patients with PAF were older (70.9 ± 8.0 vs. 62.3 ± 10.3, *p* < 0.001) and had shorter LTL (1.2 ± 0.3 vs. 1.5 ± 0.3, *p* < 0.001) than those who did not. The receiver operating characteristic (ROC) curve analysis showed a significant value of LTL in distinguishing patients with PAF from patients with PsAF, with an area under the ROC curve (AUC) of 0.63 (95% CI 0.56–0.70, *p* < 0.001), and the optimal cut-off value of LTL was 1.175, with a sensitivity and specificity of 56.03 and 82.04%, respectively. All patients with PAF were divided into two subgroups according to the optimal cut-off point of LTL calculated by the ROC curve analysis: high LTL group (≥1.175) and low LTL group (<1.175). Kaplan-Meier curve analysis showed that PAF patients with shorter LTL had a significantly higher rate of progression after catheter ablation (40.5% vs. 18.8%, log-rank test *p* < 0.001). Multivariate Cox proportional-hazards model indicated that LTL [hazard ratio (HR): 2.71, 95% CI 1.36–5.42, *p* = 0.005] was an independent predictor for progression from PAF to PsAF after catheter ablation therapy, but HATCH score was not (HR: 1.02, 95% CI: 0.68–1.52, *p* = 0.923).

**Conclusion:**

Leukocyte telomere length was significantly associated with AF types. LTL was independently associated with progression from PAF to PsAF after catheter ablation therapy.

**Chinese Clinical Trial Registry, Registration Number:** ChiCTR1900021341.

## Introduction

Atrial fibrillation (AF) is the most common sustained tachycardia in clinical practice (1). AF is significantly correlated with increased adverse cardiovascular events, including heart failure (HF), stroke, myocardial infarction (MI), all-cause death, and decreased quality of life (2, 3). Advanced age is currently recognized as the most important risk factor for the incidence of AF. The AF prevalence was merely <1% in subjects younger than 55-year old and significantly increased to ~10% in subjects over 80-year old (4, 5). With the development of aging worldwide, it is estimated that the total subjects with AF would be double in the year 2050 than the present (6, 7).

The natural disease history of AF often starts with a short and self-terminating paroxysmal AF (PAF) status and gradually transforms into a longer persistent AF (PsAF) type (8, 9). The factors contributing to AF occurrence have been well established, while the factors predicting AF progression from PAF to PsAF were not. However, it is thought to be more important because of the significantly worse prognosis for patients with PsAF than those patients with PAF (10, 11). Numerous risk factors, including modifiable and non-modifiable factors, have been identified to be significantly associated with AF progression, among which aging has been considered as the dominant risk factor associated with AF progression (12, 13).

Telomeres, which are located at both ends of chromosomes, are specific repeated DNA sequences [TTAGGG]n that function to prevent DNA degradation during cell replication (14). Telomere length (TL) is shortened with cell division because of the non-complete DNA replication. In addition to the influence of cell division, the shorting rate of TL, which is also termed telomere attrition, could be accelerated by several genetic and environmental factors, such as inflammation and oxidative stress (15, 16). When the TL reaches a certain point, the DNA damage response would be activated, which would lead to the upregulation of p-53 and thus cell apoptosis (17).

Catheter ablation is reported better than guideline-directed antiarrhythmic drug (AAD) therapy in delaying progression from PAF to PsAF (18, 19). Catheter ablation has been proposed as the first-line therapy for patients with PAF in restoring sinus rhythm (20). Despite the critical role of aging in perpetuating AF, the relation between TL and AF is still controversial. Our former study which enrolled 277 patients with AF indicated that shorter leukocyte telomere length (LTL) was significantly associated with AF recurrence after catheter ablation (21). In the present study, we aimed to investigate the association of LTL with AF types and determine the predictive value of LTL for the progression from PAF to PsAF after catheter ablation by a cross-sectional analysis method.

## Methods

### Study Design

This is an observational prospective cross-sectional study. We obtained informed consent from all subjects recruited in this study and approval from the local research ethics committee of the Beijing Chaoyang Hospital, Capital Medical University. The protocols applied in this study were in accordance with the ethical guidelines of the 1957 Declaration of Helsinki, as reflected in *a priori* approval by the Institution's Human Research Committee. One technician, who was blinded to both the baseline information and clinical endpoint, performed all analyses of the LTL measurement.

### Study Subjects

A total of 269 subjects with symptomatic nonvalvular AF (154 PAF and 115 PsAF, respectively) were prospectively selected for this study from June 2016 to December 2017. Subjects with AF were diagnosed by the documented AF episodes detected by 12-lead ECG or 24-h Holter monitor. Those who met any of the following conditions were excluded from this study: acute or chronic inflammation conditions, MI or stroke history (6 months), thyroid dysfunction, congenital heart disease, heart surgery history (6 months), and valvular disease (moderate-to-severe valvular stenosis). In addition, patients with PAF who underwent catheter ablation before were also excluded. In the present study, all patients with PAF received catheter ablation to restore sinus rhythm, and rhythm control [converted by catheter ablation or anti-arrhythmia drugs (AADs)] or rate control therapy was according to the clinical conditions for patients with PsAF. The details of catheter ablation procedures of the present study were described in the former study.

All peripheral blood samples were drawn into ethylenediaminetetraacetic acid (EDTA) tubes from consenting subjects at administration after overnight fasting. All whole blood samples were processed with centrifugation within 4 h and stored as blood cells at −80°C until later LTL analysis.

### DNA Extraction

We used QIAamp DNA Blood Midi Kits (Qiagen, Hilden, Germany) to manually extract the DNA from blood cells centrifugated from the peripheral whole blood samples. Erythrocyte was lysed and removed by a series of washing steps. Moreover, the remaining leukocytes were lysed, and other solubilized protein was removed by precipitation and centrifugation. DNA quality was evaluated by ultraviolet absorption at 260 nm/280 nm (Nanodrop 2000).

### LTL Measurement

The relative LTL was measured by quantitative PCR, which was modified from the method reported by Cawthon et al. (22) before. The telomere repeats (primers: forward-acactaaggtttgggtttgggtttgggtttgggttagtgt; reverse-tgttaggtatccctatccctatccctatccctatccctaaca) of a single gene (*HBG*, hemoglobin; primers: forward-cttcatccacgttcaccttg; reverse-gaggagaagtctgccgtt) in each sample were measured using quantitative real-time PCR (rt-PCT). The relative LTL was calculated as the ratio of telomere DNA repeats to single-copy gene (SCG) copies (t/s ratio), with *HBG* being designated as the SCG. All rt-PCR experiments were conducted in triplicate for each sample.

### Follow-Up Approaches

All patients with PAF were regularly followed up for 36 months in the present study. During follow-ups, patients would receive 12-lead ECG or 24-h Holter at baseline and 1, 3, 6, 12 months after discharge and every 12 months thereafter in scheduled clinical visits. If patients exhibit AF episodes, they would be monitored by a 7-d Holter. There would also be symptoms that triggered those visits that triggered by symptoms would also be recorded accordingly. Patients or their care providers were taught to appreciate their pulse to identify AT (atrial tachycardia)/AF at home as a supplementary for any suspicious AF episodes, while any self-reported AF episodes would be further determined by ECG. Besides, some of the patients were equipped with a smartwatch capable of recording pulse rate and morphology. When recorded AT/AF, by the above measures, lasted for more than 7 d and would not be converted by chosen cardioversion measures, the patients would be defined as AF progression. Progression of AF in the present study was defined as follows: patients first diagnosed as paroxysmal, with documented ECG as the baseline, become persistent during 3-year follow-up, regardless of the managing strategies. PAF was defined as self-terminating AF episodes or terminated by any cardioversions means within 7 d, including AADs and direct current cardioversion (DCC). AF episodes lasting beyond 7 d were defined as PsAF.

We also calculated the HATCH score for each patient with PAF in this study according to described before (23): 1 point for either history of hypertension, chronic obstructive pulmonary disease (COPD), or age ≥ 75 years; 2 points for combining either history of transient ischemic attack or stroke or HF.

### Statistical Analysis

Continuous variables were expressed as mean ± SD or median (25, 75th interquartile range), according to the variable distribution. Shapiro-Wilk test was used to evaluate the distribution of the continuous variables. For continuous variables, statistical differences between groups were tested by Student's *t*-test or the Mann-Whitney *U* test appropriately. Categorical variables were shown as frequencies (percentage) and tested by the chi-square test. Association between continuous variables was evaluated by Spearman correlation analysis. The receiver operating characteristic (ROC) curve was applied to evaluate the value of LTL in distinguishing subtypes of AF and the area the under curve (AUC), optimal cut-off value, sensitivity, and specificity were calculated, respectively. According to the cut-off value of LTL, all subjects with PAF were divided into two subgroups: LTL shortened group (LTL ≤ 1.175) and non-shortened LTL group (LTL > 1.175). The rate of free from AF progression was estimated by the Kaplan-Meier curve method, and any difference in free from AF progression was tested by the log-rank test. Multivariable Cox proportional hazards model analysis was used to evaluating the prognostic value of LTL on progression from PAF to PsAF. In this study, all statistical analyses were performed by SPSS software, version 24.0 (SPSS, Chicago, IL, USA), and a two-tailed *p*-value of <0.05 was considered to be statistically significant. All graphs in this study were drawn by GraphPad prism software, version 8.0.

## Results

### LTL Was Significantly Associated With AF Type

A total of 269 patients with AF were prospectively enrolled in this study (154 patients with PAF and 115 patients with PsAF, respectively). Baseline characteristic comparisons between different AF types are summarized in Table 1. Patients with PAF had significantly longer LTL than patients with PsAF (1.4 ± 0.3 vs. 1.2 ± 0.3, *p* = 0.003), while the age (64.3 ± 10.4 vs. 64.3 ± 9.1 *p* = 0.998) was comparable between the two groups. In addition, patients with PsAF had significantly increased levels of high-sensitivity C-reactive protein (hs-CRP) (2.0 vs. 1.1, *p* = 0.001), N-terminal pro-natriuretic peptide (NT-proBNP) (953.7 vs. 221.2, *p* < 0.001), left atrial diameter (LAD) (45.2 ± 5.9 vs. 39.1 ± 4.8, *p* < 0.001), and decreased level of left ventricular ejection fraction (LVEF) (60.6 ± 11.3 vs. 65.4 ± 7.2, *p* < 0.001). A higher proportion of HF (56.5% vs. 24.0%, *p* < 0.001) and stroke history (24.3% vs. 12.3%, *p* = 0.010) were also observed in a group with PsAF.

**Table 1:** Baseline characteristics by AF type.

**Variables**	**PAF (*n =* 154)**	**PsAF (*n =* 115)**	***p*-value**
Age (year)	64.3. ar 0.4	64.3 ± 9.1	0.998
Male	90(58.4%)	67(58.3%)	0.976
BMI (kg/m^2^)	25.8 ± 3.8	26.9 ± 4.3	0.023
Smoking	60(39.2%)	44(38.3%)	0.874
CAD	35(22.7%)	32(27.8)	0.339
HF	37(24.0%)	65(56.5%)	<0.001
DM	41(26.6%)	26(22.6%)	0.451
hypertension	94(61.0%)	74(64.3%)	0.579
stroke	19(12.3%)	28(24.3%)	0.010
COPD	5(3.2%)	3(2.6%)	0.761
ACEI/ARB	59(38.3%)	50(43.5%)	0.393
β-blocker	63(40.9%)	58(50.4%)	0.120
Statins	81(52.6%)	68(59.1%)	0.286
hs-CRP (mg/L)	1.1(0.6,2.5)	2.0(1.1,4.7)	<0.001
NT-rpoBNP (pg/ml)	221.2(82.3,814.4)	953.7(499.0, 1,964.0)	<0.001
eGFR (ml/min. 1.73 m^2^)	89.0 ± 16.1	85.0 ± 17.7	0.054
t/s ratio	1.4 ± 0.3	1.2 ± 0.3	0.002
LAD (mm)	39.1 ± 4.8	45.2 ± 5.9	<0.001
LVEF (%)	65.4 ± 7.2	60.6 ± 11.3	<0.001

*BMI, body mass index; CAD, coronary artery disease; HF, heart failure; DM, diabetes mellitus; COPD, chronic obstructive pulmonary disease; ACEI, angiotensin-converting enzyme inhibitors; ARB, angiotensin receptor blocker; hs-CRP, high-sensitivity C-reactive protein; NT-proBNP, N-terminal pronatriuretic peptide; e-GFR, estimated glomerular filtration rate; t/s ratio, the ratio of telomere repeats to a single-copy gene (SCG) copies; LAD, left atrial diameter; LVEF, left ventricular ejection fraction*.

**Figure 1:**
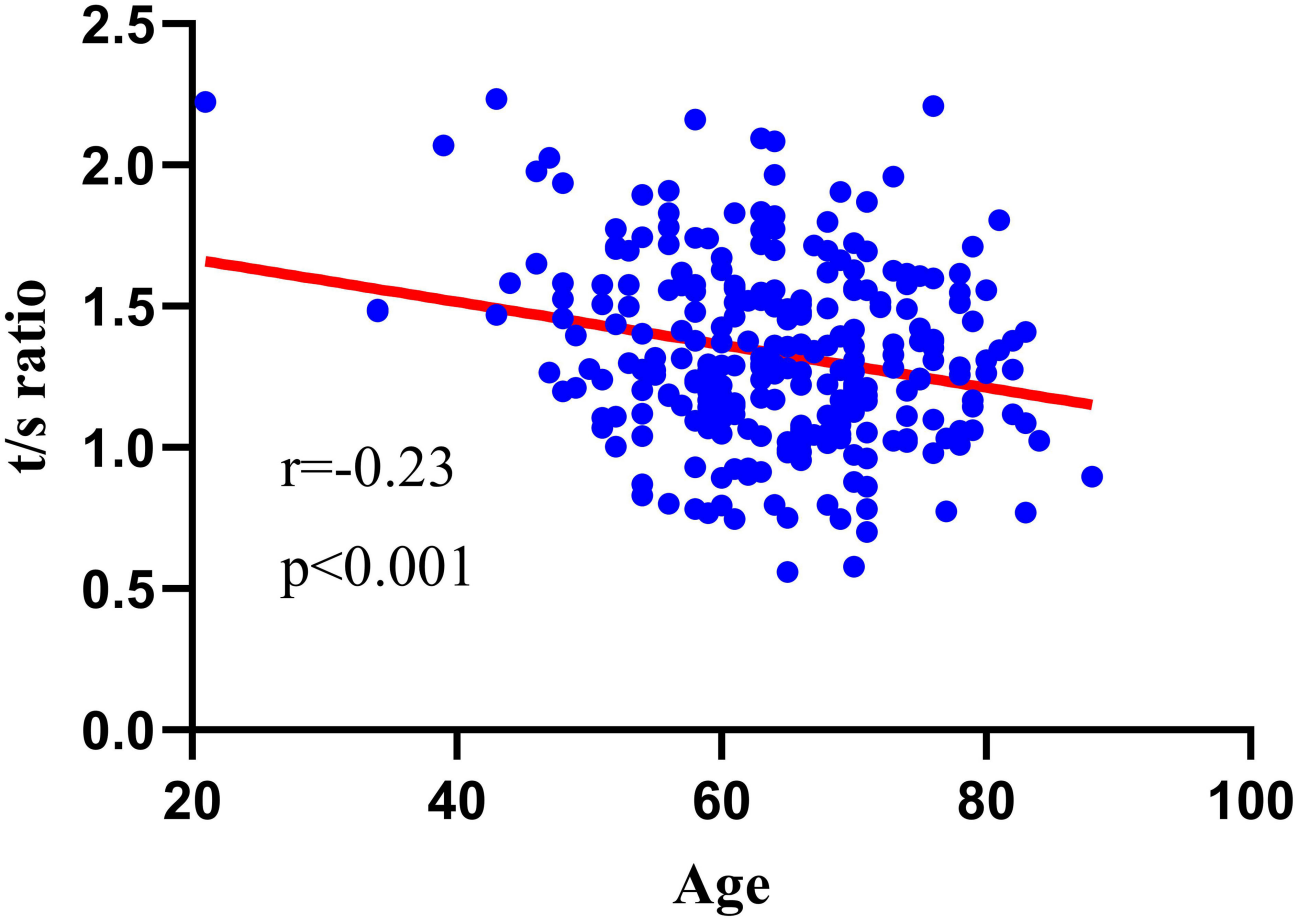
Relationship between LTL and age. TLT was negatively associated with advanced age (*r* = −0.23, *p* < 0.001).

Spearman correlation analysis was used to evaluate the association between age and LTL, and the result is as shown in Figure 1. Advanced age was negatively associated with LTL (*r* = −0.23, *p* < 0.001).

### Shorter LTL Was Significantly Associated With PAF Progression

Baseline characteristics comparison between patients with PAF with and without progression are as shown in Table 2. During a median follow-up of 854.9 ± 18.7 days, AF progression occurred in 35 (22.7%) out of 154 patients with PAF who were successfully followed up. Patients with AF progression were older (70.9 ± 8.0 vs. 62.3 ± 10.3 *p* < 0.001), had significantly increased levels of NT-proBNP (484.5 vs. 183.6 *p* = 0.004), HATCH score (2 vs. 1 *p* = 0.002), and LAD (40.7 ± 4.8 vs. 38.7 ± 4.7 *p* = 0.028), and decreased level of e-GFR (80.5 ± 16.8 vs. 91.5 ± 15.1 *p* < 0.001) and LTL (1.2 ± 0.3 vs. 1.5 ± 0.3 *p* < 0.001), and higher proportion of HF (38.2% vs. 20.2% *p* = 0.039) and COPD history (8.6% vs 1.7% *p* = 0.043) than those who did not progress.

**Table 2:** Baseline characteristics by AF progression.

**variables**	**Progression (*n =* 35)**	**No progressions (*n =* 119)**	***p*-value**
Age (year)	70.9. ru .0	62.3. ru 0.3	<0.001
Male	26(59.1%)	88(58.3%)	0.923
BMI (kg/m^2^)	25.7 ± 3.9	25.7 ± 3.7	0.995
Smoking	13(38.2%)	47(39.5%)	0.894
CAD	7(20.0%)	28(23.5)	0.661
HF	13(37.1%)	24(20.2%)	0.039
DM	12(34.3%)	29(24.4%)	0.243
Hypertension	24(68.6%)	70(58.8%)	0.299
Stroke	5(14.3%)	14(11.8%)	0.690
COPD	3(8.6%)	2(1.7%)	0.043
ACEI/ARB	14(40.0%)	45(37.8%)	0.815
β-blocker	17(48.6%)	46(38.7%)	0.294
Statins	15(42.9%)	66(55.5%)	0.189
hs-CRP (mg/L)	1.3(0.7,3.5)	1.1(0.6,2.0)	0.197
NT-proBNP (pg/ml)	484.5(116.9,1624.0)	183.6(69.4,680.2)	0.004
eGFR(ml/min. 1.73 m^2^)	80.5 ± 16.8	91.5 ± 15.1	<0.001
t/s ratio	1.2 ± 0.3	1.4 ± 0.3	<0.001
HATCH score	2(1,3)	1(0,2)	0.002
LAD (mm)	40.7 ± 4.8	38.7 ± 4.7	0.028
LVEF (%)	66.5 ± 8.0	65.1 ± 7.0	0.302

*BMI, body mass index; CAD, coronary artery disease; HF, heart failure, DM, diabetes mellitus; COPD, chronic obstructive pulmonary disease; ACEI, Angiotensin-Converting Enzyme Inhibitors; ARB, angiotensin receptor blocker; hs-CRP, high-sensitivity C-reactive protein; NT-proBNP, N-terminal pronatriuretic peptide; e-GFR, estimated glomerular filtration rate; t/s ratio, the ratio of telomere repeats to a single-copy gene (SCG) copies; LAD, left atrial diameter; LVEF, left ventricular ejection fraction*.

### Shorter LTL Was a Potential Risk Factor for Progression From PAF to PsAF

**Figure 2:**
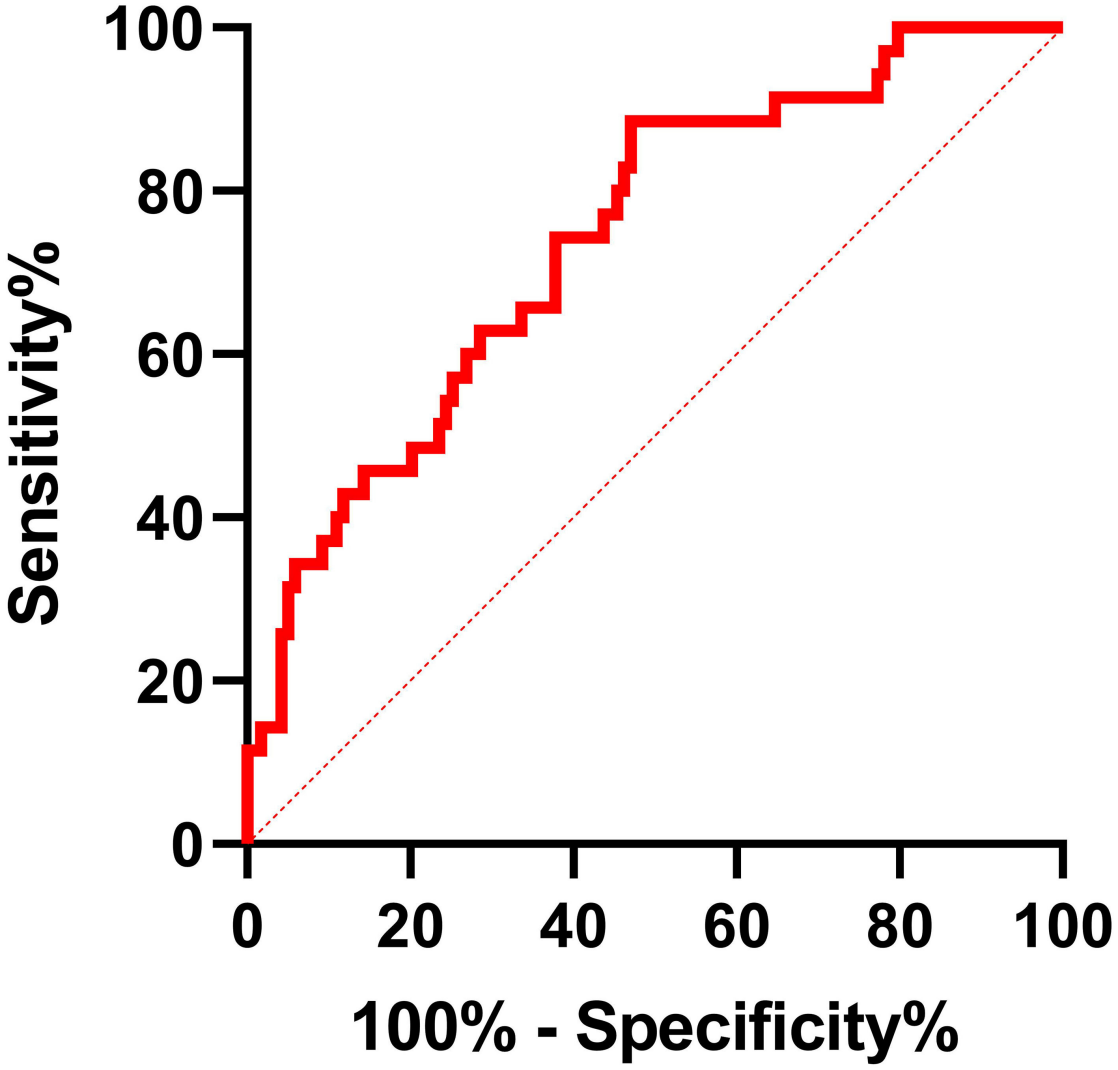
ROC curve analysis for LTL in distinguishing PsAF patients from patients with PAF. AUC of shorter LTL was 0.63 (95% CI: 0.56–0.70, *p* < 0.001), with sensitivity and specificity of 56.03% and 82.04%, respectively. The optimum cut-off value for LTL was 1.175.

**Figure 3:**
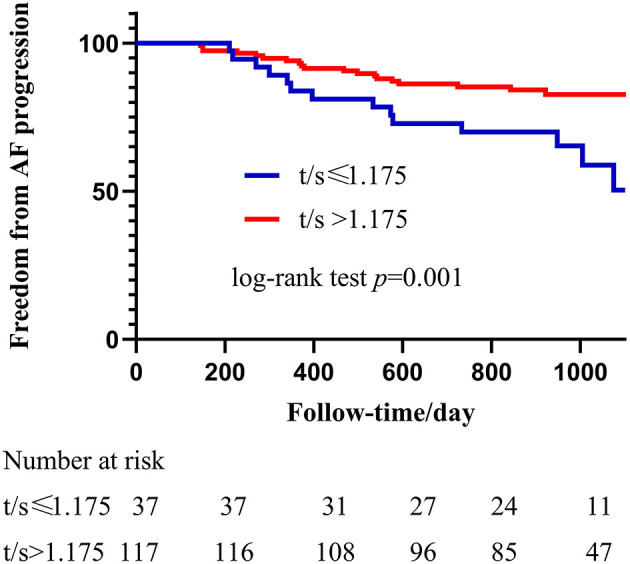
Kaplan-Meier analysis of Shorter LTL in predicting progression from PAF to PsAF after catheter ablation. The blue line represents LTL shorter or equal to 1.175 and the red line represents LTL longer than 1.175. Progression rate in shortened LTL patients was significantly higher than non-shortened, and HR = 2,71 (95% CI: 1.36–5.42, *p* = 0.005).

A receiver operating characteristic curve was used in this study to evaluate the value of LTL in distinguishing patients with PsAF from patients with PAF. As shown in Figure 2, LTL shows a significant diagnostic value for distinguishing patients with PsAF from patients with PAF, with AUC = 0.63 (95% CI: 0.56–0.70), and the optimal cut-off point was calculated as 1.175 with sensitivity and specificity of 56.03 and 82.04%, respectively. All patients with PAF were divided into two subgroups according to the optimal cut-off point calculated by the ROC curve: shorten LTL group (LTL ≤ 1.175) and non-shorten LTL group (LTL > 1.175). Kaplan-Meier curve analysis was used to estimate the difference of the cumulative proportional probabilities of the occurrence of progression between the two groups, as shown in Figure 3. Patients with shorter LTL had a significantly increased cumulative probability of AF progression than those with longer LTL (55.3% vs. 14.6%, log-rank test *p* < 0.001).

### Shorter LTL Was an Independent Predictor for Progression From PAF to PsAF

A Cox proportion hazard model by the stepwise forward method was used to determine the risk factors for predicting progression from PAF to PsAF and results are shown in Table 3. Multivariate Cox regression analysis showed that shorter LTL (HR 2.71, 95% CI 1.36–5.42, *p* = 0.005), advanced age (HR 1.06, 95% CI 1.01–1.13, *p* = 0.043), COPD (HR 4.29, 95% CI 1.14–16.15, *p* = 0.031), and LAD (HR 1.09, 95% CI 1.00–1.19, *p* = 0.047) were independent predictors for progression from PAF to PsAF, even after adjustment of other confounding risk factors.

**Table 3:** Cox regression analysis for progression of AF.

**variables**	**Univariate**		**Multivariate**	
	**HR (95% CI)**	***p*-value**	**HR (95%CI)**	***p*-value**
t/s ratio	2.87 (1.48–5.59)	0.002	2.71(1.36–5.42)	0.005
age	1.09(1.05–1.13)	<0.001	1.06(1.01–1.13)	0.043
HF	2.13(1.07–4.24)	0.032	1.16(0.37–3.67)	0.800
COPD	4.89(1.48–16.11)	0.009	4.29(1.14–16.15)	0.031
stroke	1.17(0.45,3.03)	0.744		
NT-proBNP	1.00(1.00–1.00)	<0.001		
LAD	1.10(1.02–1.18)	0.017	1.09(1.00–1.19)	0.047
e-GFR	0.97(0.95–0.98)	<0.001	0.99(0.96–1.02)	0.413
HATCH score	1.36(1.11, 1.66)	0.003	1.02(0.68–1.52)	0.923

*HR, hazard ratio; CI, confidence interval; t/s ratio, the ratio of telomere repeats to a single-copy gene (SCG) copies; HF, heart failure; COPD, chronic obstructive pulmonary disease; NT-proBNP, N-terminal pronatriuretic peptide; LAD, left atrial diameter; e-GFR, estimated glomerular filtration rate*.

## Discussion

In this study, we investigated the predictive value of LTL for progression from PAF to PsAF after catheter ablation therapy. The main findings in the present study were as follows: (1) LTLs were significantly associated with AF types; (2) LTLs were significantly shorter in patients with AF progression than those without; (3) LTL was an independent predictor for progression from PAF to PsAF, even after adjustments with other confounding risk factors.

In the present study, we found a significantly shorter LTL in the PsAF group than in the PAF group. Carlquist et al. (24) found that in a cross-sectional cohort, shorter LTL was significantly associated with AF, even after adjusting for age and other risk factors. However, in the further analysis of AF subtype, just PAF, not PsAF, was significantly associated with LTL. In addition, LTL was shorter in patients with PAF than sinus rhythm (SR) controls, patients with PsAF, and even permanent AF individuals. The possible explanation for the results in the Carlquist et al. was the study population selection. The subjects in that study came from a population who underwent angiography and the majority of the subjects had coronary heart disease. In fact, in Carlquist et al.'s study, the rate of CAD in subjects with PAF was significantly higher than that in patients with PsAF or permanent AF (82.7% vs. 78.5% vs. 65.1, *p* = 0.01). Previous studies have found a significant association between shorter TL and CAD, which indicates that CAD may influence the TL. Thus, the selection bias in Carlquist et al's study may affect the findings. Besides, by following up on the patients with PAF, we have also found that shorter LTL was independently associated with PAF progression from PAF to PsAF. This could in part underlie the association of worse AF phenotype (in this study, the propensity to PsAF, not to say the worse PsAF and Permanent AF) with shorter LTL. However, further studies are still in need to render more pieces of evidence for either of the conclusions.

The rate of AF progression in our present study was 8.4% (13/154) in the first year of follow-up and the overall rate was 22.7% (35/154), which was similar to a previous study constructed by Gareth et al. in the Canadian Registry of AF (13). The rate of AF progression in the present study was higher than the study constructed by Cees et al. (23), who also developed the HATCH score, which was used to predict progression from PAF to PsAF in the clinical context. The discrepancies of progression rates between their study and ours may be explained by the length of follow-up: the follow-up periods of the present study last for 3 years, which is longer than 1 year in the study of Cees et al. Besides, the progression rate in the present study in 1 year was lower than their study, perhaps due to the different strategy of restoring SR: patients with PAF were restored by catheter ablation in this study, while all patients were restored by AADs in that study.

In this study, we also investigated the value of the HATCH score for predicting progression from PAF to PsAF. We found that those patients with PAF who progressed to PsAF status indeed had higher HATCH scores than those who did not, however, the HATCH score was not an independent predictor for AF progression in this study (HR 0.89, 95% CI: 0.58–1.37, *p* = 0.597). Jongnarangsin et al. (25) have evaluated the predictive value of HATCH score in AF progression after catheter ablation and revealed that HATCH score was not an independent predictor for AF progression after ablation therapy. Tang et al. (26) also reported that the HATCH score was not independently associated with AF recurrence after catheter ablation.

Inflammation is one of the most important risk factors for accelerating telomere attrition and promoting the aging process. In total, 15–20 cell divisions with naive T cells may be involved in an immune response and lead to telomere loss. Jurk et al. (27) demonstrated that an nfkb1^−^/^−^ mice model with chronic inflammation conditions significantly upregulated the reactive oxygen species (ROS) and accelerated telomere shorting. Besides, Amsellem et al. (28) observed a comparable pro-inflammatory state in late-generation *Terc*^−^/^−^ and *Tert*^−^/^−^ mice models that were used for telomere dysfunction-driven senescence analysis, which was often seen in late chronic inflammation conditions.

Recent studies have found that shortening TL was significantly associated with various tissue fibrosis, including pulmonary fibrosis (29), liver fibrosis (30), and renal fibrosis (31). Despite the fact that there was no current evidence indicating the association of shorter TL with cardiac fibrosis, a recent study that investigated the mechanisms between TL and pulmonary fibrosis may provide some insights for us. Ying-Ying Liu et al. (32) found that Terc^−^/^−^ mice with shorter telomeres developed significantly aggravated pulmonary fibrosis than wide-type mice with normal telomeres. In addition, TGF-β/Smad signaling, which plays a key role in promoting tissue fibrosis, was also markedly activated in the lungs of G3 Terc^−^/^−^ mice, indicating that shorting telomeres might enhance fibrosis via activation of TGF-β/Smads signaling, which is also the key pathway in cardiac fibrosis and AF persistency (33).

Catheter ablation therapy has been proved more effective in maintaining sinus rhythm and delaying AF progression than AADs (18, 19). A recent meta-analysis indicated that compared to the medication rate-control therapy, catheter ablation therapy significantly improves cardiac function evaluated by LVEF, exercise capacity, and quality of life (34). Thus, catheter ablation was proposed as the first-line strategy to restore sinus rhythm for patients with AF, especially for those patients with PAF in recent years. Kuo-Li Pan et al. found that shorter LTL was significantly associated with cardiac remodeling and AF recurrence after catheter ablation in younger patients ( ≤ 55 years) (35). A recent study found that shorter LTL was an independent predictor for progression from PAF into PsAF (36). In this study, we confirmed that shorter LTL was independently associated with progression from PAF to PsAF. The main discrepancies between their study and this study were the intervention means of restoring sinus rhythm. Only 35.3% (42/119) of patients with PAF had accepted catheter ablation in that study, while all patients with PAF had received catheter ablation therapy. In addition, the rate of progression in that study was relatively low than our study (16.8% vs. 22.7%), which may be due to the shorter length of follow-up (18 months vs. 36 months).

## Limitations

Several limitations should be mentioned in this study. First, although we did find a significant association between LTL and AF progression, we could not obtain a direct causal-effect relationship. Second, in our study, the relative LTL length was measured based on an rt-PCR analysis instead of Southern blotting, which has been the gold standard in LTL measuring. However, the relative length of LTL has long been a recognized mean in determining LTL/disease association studies. It may be necessary to utilize the absolute length of LTL as measured by Southern blotting in clinical settings, but this did not undermine the conclusions of the present study. Third, we did not obtain direct evidence of cardiac fibrosis, further studies are still needed. Finally, self-reported AF episodes may be inaccurate, which may affect the progression rate. Thus, all self-reported AF episodes were requested to confirm by ECG examination in this study.

## Conclusion

Leukocyte telomere length was significantly associated with AF type, and patients with PsAF had significantly shorter LTL than patients with PAF. LTL was an independent predictor for progression from PAF to PsAF after catheter ablation.

## Data Availability Statement

The raw data supporting the conclusions of this article will be made available by the authors, without undue reservation.

## Ethics Statement

The studies involving human participants were reviewed and approved by Ethics Committee of Beijing Chaoyang Hospital, Capital Medical University. The patients/participants provided their written informed consent to participate in this study.

## Author Contributions

MC and YG: designed the study. QW: collected the data. YD and QW: validated and inspected the data and performed the statistical analysis. QW, ZL, and YG: wrote the manuscript. MC, XY, and YG: revised the manuscript. All authors contributed to the article and approved the submitted version.

## Funding

The present study was supported by the Beijing Hospitals Authority Youth Programme, code: QML20210301; Beijing Hospitals Authority Clinical medicine Development of special funding support, code: XMLX202135; the National Natural Science Foundation of China (81700295); the open funding for Key Laboratory of Medical Engineering in Cardiovascular Disease Research, Ministry of Education (2020XXG-KFKT-02).

## Conflict of Interest

The authors declare that the research was conducted in the absence of any commercial or financial relationships that could be construed as a potential conflict of interest.

## Publisher's Note

All claims expressed in this article are solely those of the authors and do not necessarily represent those of their affiliated organizations, or those of the publisher, the editors and the reviewers. Any product that may be evaluated in this article, or claim that may be made by its manufacturer, is not guaranteed or endorsed by the publisher.
